# The use of informal care by people with vision impairment

**DOI:** 10.1371/journal.pone.0198631

**Published:** 2018-06-07

**Authors:** Ana Patricia Marques, Antonio Filipe Macedo, Laura Hernandez-Moreno, Pedro Lima Ramos, Thomas Butt, Gary Rubin, Rui Santana, for the Portuguese visual impairment study group (PORVIS-group)

**Affiliations:** 1 National School of Public Health, NOVA University of Lisbon, Lisbon, Portugal; 2 Public Health Research Center, National School of Public Health, NOVA University of Lisbon, Lisbon, Portugal; 3 Department of Medicine and Optometry Linnaeus University Kalmar, Kalmar, Sweden; 4 Low Vision and Visual Rehabilitation Lab, Department and Center of Physics—Optometry and Vision Science, University of Minho Braga, Braga, Portugal; 5 University College London, Institute of Ophthalmology, London, United Kingdom; 6 National School of Development, Peking University, Beijing, China; Institut de la vision, FRANCE

## Abstract

**Purpose:**

To estimate and characterize the use of informal care by people with vision impairment in Portugal.

**Methods:**

A total of 546 visually impaired individuals were recruited from Portuguese hospitals. Clinical information was obtained from medical records, socio-demographic details and informal care use were collected during face-to-face interviews. In addition, participants responded to a functional vision questionnaire (activity inventory) to assess their visual ability. Logistic regression was used to determine independent factors associated with informal care use and linear regression was used to determine independent predictors of intensity of informal care use.

**Results:**

Informal care was reported by 39.6% of the participants. The probability of reporting informal care was higher in non-married, those with comorbidities, with lower visual ability and worse visual acuity. The median number of caregivers’ hours per year was 390 (mean = 470; 95%CI = 488–407), which represent a median opportunity cost of €2,586. Visual ability was the only independent predictor of number of hours of informal care received.

**Conclusions:**

Informal care was frequently used by individuals with impaired vision. Improving visual ability of people with impaired vision when performing valued activities may reduce the burden of visual loss at personal and societal level. This could be achieved with person-centred visual rehabilitation.

## Introduction

Vision impairment puts a burden on individuals, families and society. People with impaired vision require more informal care to perform activities of daily living, have more difficulties with mobility, have increased risk of falls and are more likely to require long term care than persons without vision impairment [1–4]. Informal care is generally defined as “help provided to older and dependent persons by non-professional individuals such as, a spouse, parent, other relative, neighbour or friend, in a wide variety of activities and with no payment associated” [5, 6]. Some factors such as age, type of activities, type of disability and severity level can influence the demand for informal care [5, 7]. In addition, it can be influenced by socio-cultural aspects such as family structures, levels of income per capita and availability of formal long-term care systems [5]. In 2007 the Portuguese minister of health implemented a national network of integrated care to provide health and social support including long term care. Visually impaired persons may have access to the national network of integrated care when they meet the access criteria. Nevertheless, access is difficult due to the limited capacity of the network and in some cases, due to the co-payment associated. Therefore, in Portugal, long-term care for people with impaired vision remains mostly informal, that is, provided by relatives or friends.

Informal care tends to be a major contributor to the total costs of vision impairment [8]. Some studies investigated informal care costs in people with impaired vision due to specific eyes diseases such as age-related macular degeneration or diabetic retinopathy [9–11] considering, in a few instances, self-reported difficulties [9, 10]. However, one study relied in presumed visual acuity [9] and another failed to investigate the effect of self-reported difficulties in informal care [10]. Other authors reported the use of informal care by people with impaired vision but did not used structured and validated questionnaires to assess limitations with daily activities. Although, some took in consideration limitation to mobility imposed by vision impairment [12, 13]. In general, self-reported difficulties have been overlooked in past studies so further research is needed to characterize the use of informal care and its predictors in this population.

The aims of this study were to estimate and characterize informal care use in persons with impaired vision in Portugal and to investigate the association between informal care, clinical and socio-demographic aspects. We used a bottom-up approach and administered validated questionnaires to a sample of people with impaired vision.

## Methods

### Study design, setting and participant selection

Participants were recruited from 4 public hospitals with ophthalmology departments in the north of Portugal between July 2014 and January 2016. Outpatients at these hospitals with a latest recorded visual acuity of 0.30 logMar (6/12) or worse in the better seeing eye were invited to take part in face-to-face interviews. Patients were invited by letter posted using the hospital mail service, the logo of the hospital was printed on the envelope and letters went sent directly to the patients`address. All documents were printed in font Arial– 16 point. The mail envelope include a letter of invitation signed by a physician from the local hospital, an information booklet and a consent form. Despite some letters were returned to sender due to incorrect address, we estimate that at least 3000 reached the patient´s home, 546 returned a signed consent form on a reply-paid envelope addressed to Escola Nacional de Saúde Pública, Lisboa (National School of Public Health, Lisbon) with an updated phone number. After acceptance participants were contacted and an interview was scheduled at the hospital.

Causes of visual impairment, principal diagnosis and secondary diagnosis, were retrieved from clinical records and classified according with the ICD9 MC (International Classification of Diseases 9th Clinical Modification) codes. The information was registered in a secure platform that is online at www.pcdvp.org.

This study has been designed according to guidelines published by the Vancouver Economic Burden of Vision Loss Group [14]. The study was conducted in accordance with the tenets of the Declaration of Helsinki, reviewed and approved by the ethical committee for Life Sciences and Health of the University of Minho and local ethics committees at Centro Hospitalar São João, Hospital de Braga, Centro Hospitalar do Alto Ave and Hospital de Santa Maria Maior. Written informed consent was obtained from all participants. Further details about the study have been described in our previous publications [15].

### Clinical measurements during face-to-face interviews

Patients answered a functional vision questionnaire, the Activity Inventory (AI), to assess visual ability. The AI is an adaptive visual function questionnaire designed to provide an individualized assessment of difficulties of a visually impaired respondent when performing valued activities. Disabilities, or activity limitations according to the World Health Organization’s International Classification of Functioning, occur when an individual reports abnormal difficulties in achieving important goals. Difficulties achieving a goal are said to depend on the difficulty experienced in the tasks that underlie each goal [16–19]. In our translated version of the Massof activity inventory patients were questioned about difficulties with 46 goals and the “difficulty” responses were Rasch analysed to produce a continuous measure of visual ability given by the variable ‘person measure’ (Program Winsteps, v3.9) [18, 20]. We use the term ‘visual ability’ to define the overall ability to perform activities that depend on vision [21].

Participants also reported comorbidities based on a list of 16 categories as described in S1 Appendix. Visual acuity with the habitual correction was re-assessed in both eyes separately using an internally illuminated ETDRS chart (Lighthouse International, NY, USA) at 4, 2 or 1 meter–the measuring distance was adjusted according with the severity of the expected vision loss. The room lights where extinguished during measurements. Letter by letter scoring was employed to specify the final measured acuity [15].

### Informal care questionnaire and cost estimation

A questionnaire to collect information about informal care was administered by trained researchers. We asked information about the use of informal care within a 2-week recall time period. This period has been proposed by others to minimise recall biases [22, 23]. The questionnaire was drawn from previously validated instruments [23, 24], it underwent pilot testing and revisions to clarify wording, to simplify data recording and to remove redundant items. The final version of the questionnaire is summarized in Table 1.

**Table 1:** The table summarises the questions used to collect information about sociodemographic information and the use of informal care. In all questions, there was one option with: *do not know or do not want to answer* (not shown in the table for simplicity).

Categories	Questions	Options
Sociodemographic information	1. What is your marital status?	a) Single b) Married or living as married c) Divorced or separated d) Widowed e) Other (please specify)
	2. What best describes your living environment?	a) Live alone b) Live with spouse (including children) c) Live with parents d) Live with children (sons / daughters) e) Live with other relatives f) Live with others
Informal Care Need	3. Over the last two weeks have you been helped and/or cared for by a relative or friend because you have poor vision?	a) Yes (ask question 3.1)
		b) No
	3.1. Over the last two weeks how many relatives or friends provided you care?	Number of caregivers
	3.2. Over the last two weeks how many hours were spend by caregivers to help you?	Number of hours
	3.3 What is the main occupation of the persons providing you care?	a) Paid job b) Currently seeking work c) Homemaker d) Retired e) Student f) Other (please specify)

Informal care costs represent a monetary estimate of the hours spend by informal caregivers to help visually impaired persons. To estimate the economic impact of informal care we used the opportunity costs in which time spent providing informal care is valued based on competing time use, in this case paid labour. This method is commonly used for these estimates including in eye care and rehabilitation studies [5, 10, 25, 26]. The number of reported hours were extrapolated from 2 weeks to 12 months. Total informal care costs were calculated by multiplying the number of annual hours by the mean Portuguese hourly wage rate of full time employees in the year 2014 (€6.63). This value is within the interval €4.10 to €19.18 reported by Costa et al. [27].

### Statistical analysis

Descriptive statistics was used to summarize socio-demographic and clinical characteristics of the participants. Participants were divided into 4 age categories: (1) 39 years or younger; (2) 40–64 years; (3) 65–79 years; (4) 80 years and older. Causes of visual impairment were divided in 11 categories (see Table 2). Visual acuity was used either as continuous variable or categorical variable whichever was deemed more appropriate.

**Table 2:** The socio-demographic and clinical characteristics of study participants (n = 546).

	Informal Care				
	Users n = 216		Non-users n = 330		Group comparison
	n	(%)	n	(%)	
**Gender**					Chi-square = 6.3; **p = 0.012**
Female	120	56%	147	45%	
Male	96	44%	183	55%	
**Age categories (years)**					Chi-square = 1.36; p = 0.714
39 years or younger	16	7%	32	10%	
40 to 64	92	43%	131	40%	
65 to 79	85	39%	136	41%	
80 or older	23	11%	31	9%	
**Marital status**					Chi-Square = 4.3; **p = 0.037**
Not Married	91	42%	110	33%	
Married	125	58%	220	67%	
**Living arrangement**					Chi-square = 0.13; p = 0.724
Live alone	25	12%	35	11%	
Live with others	191	88%	295	89%	
**Cause of Visual impairment (principal diagnosis)**					Chi-square = 12.9; p = 0.228
Diabetic Retinopathy	82	38%	115	35%	
AMD	20	9%	44	13%	
Glaucoma	27	13%	32	10%	
Other Retinal Disorders and Detachments	25	12%	27	8%	
Cornea	13	6%	28	8%	
High Myopia	13	6%	28	8%	
Cataracts	4	2%	15	5%	
Disorders of Choroid	9	4%	8	2%	
Optic Nerve Disorders	9	4%	10	3%	
Disorders of Globe	5	2%	4	1%	
Others	9	4%	19	6%	
**Secondary diagnosis**					Chi-square = 1.7; p = 0.194
Yes	117	54%	160	48%	
No	99	46%	170	52%	
**Other Comorbidities**					Chi-square = 6.0; **p = 0.014**
Yes	164	76%	218	66%	
No	52	24%	112	34%	

Chi-square tests were used to compare the composition of groups. T-tests were used to compare visual ability between groups and the Mann-Whitney–test or Kruskal-Wallis were used for other non-parametric comparisons between groups. Spearman correlation was used to determine the association between variables.

Logistic regression was used to determine explanatory factors associated with the use of informal care. Linear regression was used to determine factors associated with the amount of informal care needed (intensity of care). A description of the models is provided in S2 Appendix. Statistical analysis was performed with SPSS Statistics (IBM SPSS Statistics v.23).

## Results

A total of 546 participants were included in this study, from those 216 (39.6%) reported informal care needs. The sample comprised a high percentage of older individuals, 50% (n = 275) were older than 65 years. The most common causes of vision impairment were: diabetic retinopathy, age-related macular degeneration (AMD), glaucoma and other retinal disorders and detachments. Participants were divided in 2 groups: “users” and “non-users” to identify sociodemographic and clinical independent predictors of the use of informal care. We compared the distribution of cases, between groups, according to several categories. These results are summarized in Table 2. The proportion of women and participants with comorbidities was higher in the users group. The percentage of married individuals was higher in the non-users group.

Table 3 summarizes and compares visual ability and visual acuity in informal care users and non-users. Visual ability and visual acuity (both in the better eye and in the worse eye) were worse in the users group.

**Table 3:** Visual ability (person measures) and distance visual acuity of users and non-users of informal care in the sample. SD = standard deviation; IQR = Inter-quartile range.

	Informal care				Group Comparison	
	Users		Non-users			
Visual ability (logits)						
Mean (SD)	-0.51 (1.48)		1.6 (2.01)		t-test = 14.1; **p<0.001**	
Median (IQR)	-0.52 (1.83)		1.42 (2.95)			
Visual acuity (logMar)^a^						
	Better eye	Worse eye	Better eye	Worse eye	Better eye	Worse eye
Mean (SD)	0.87 (0.61)	1.55 (0.82)	0.47 (0.32)	0.97 (0.72)	z-test = 9.11; **p<0.001**	z-test = 8.74; **p<0.001**

^a^ In logMar scale higher values of acuity correspond to higher level of impairment

Table 4 summarizes the results of a logistic regression to determine predictors of the use of informal care. Marital status, comorbidities, visual acuity, and visual ability were independent predictors of the use of informal care. When the odds ratio (OR) reported in Table 4 is less than 1, the reciprocal is used here in the text for consistency of interpretation. Non-married individuals were 1.85 times more likely to use informal care. Individuals with comorbidities were 2.17 times more likely to use informal care than those without. An additional unit of visual acuity in the better eye increases the odds of using informal care 3.2 times (1 unit of visual acuity = 1 LogMar; higher values of acuity correspond to higher level of impairment). One unit reduction in visual ability increase the odds of using informal care 2.22 times (1 unit of visual ability = 1logit; lower values of visual ability are associated with increased difficulty to perform tasks that rely on vision). The deviance chi-squared goodness (residual deviance = 463.706; 524 degrees-of-freedom) of fit test confirmed an excellent fit of the model, p = 0.96.

**Table 4:** Explanatory variables of informal care usage. In the first column, brackets show dummy variables categories (category = 1 versus reference category = 0).

Explanatory variables	Odds Ratio	95% C.I. for Odds Ratio		p-value
		Lower	Upper	
Gender (Male *versus* Female)	0.87	0.55	1.38	0.545
Marital Status (not Married *versus* Married)	1.85	1.14	2.99	0.013
Presence of other comorbidities (No *versus* Yes)	0.46	0.27	0.77	0.003
Visual Acuity	3.2	1.71	6	<0.001
Visual Ability	0.45	0.37	0.55	<0.001

Among those who needed informal care, 60% reported having only one caregiver and the main activity of the caregivers was homemaker (the person whose principal role is to do housework and other domestic concerns). The median number of caregiver hours was 390 hours per year and the mean number of caregivers’ hours was 470 hours per year (95%CI = 488.1–406.6) which represent a median cost of €2,585.7 per year. Therefore, in our sample of 206 cases (10 out of the initial 216 cases were considered outliers) that would correspond to 92,144 hours of informal care per year, resulting in an annual cost of €610,915.0.

The number of caregivers’ hours was statistically different between categories of vision impairment (Kruskal-Wallis = 10.86; p-value = 0.012). Categories: 1) visual acuity from 0.3 logMar to 0.5 logMar; 2) visual acuity from 0.51 logMar to 1.0 logMar; 3) visual acuity from 1.02 logMar to 1.3 logMar and 4) visual acuity from 1.32 logMar and 3.0 LogMar. The pairwise comparison showed that participants in categories 1 and 2 needed less informal care than participants in categories 3 and 4 (Mann-Whitney U = 3200; p = 0.002). These results show that the use of informal care tends to increase with the severity of vision impairment. Differences between groups according with gender, causes of vision impairment, presence of comorbidities, marital status and living arrangement were not statistically significant.

There was a negative association between visual ability and the amount of informal care used, Spearman´s rho = -0.381(p<0.001). This means that lower visual ability was associated with increased use of informal care (intensity). Table 5 summarizes results of the multiple linear regression analysis used to investigate predictors of the intensity of use of informal care. Visual ability was the only statistically significant independent predictor (p<0.001). These results show that a one-unit change in visual ability corresponds, per year, to a variation of 67 hours in the intensity of informal care. The model also includes as explanatory variables age, gender and severity of vision impairment (2 categories) that were not statistically significant. The R-squared of the model was 0.157.

**Table 5:** Explanatory variables of intensity of use of informal care. In the first column brackets show the reference categories.

Explanatory variables	Unstandardized Coefficients		p-value
	B	Std. Error	
Visual ability	-66.99	14.81	<0.001
Age	0.93	1.28	0.468
Gender (male)	48.70	38.64	0.209
Severity of Visual Impairment (visual acuity in the better eye above 1 logMar)	74.79	46.75	0.111

## Discussion

In this study, we quantified and characterized the use of informal care in a sample of 546 individuals with impaired vision. Informal care was reported by 39.6% of the participants requiring, each requiring a median of 390 hours of informal care per year. Based on the median values, that corresponds to an estimated 92,000 hours per year for our 216 users. The use of informal care was influenced by marital status, comorbidities, visual ability and acuity. The intensity of use of informal care was negatively associated with visual acuity. However, lower visual ability was the only predictor of higher informal care utilisation intensity after controlling for age, gender and severity of vision impairment.

The percentage of informal care users found in our study is similar to other studies [9, 12]. One study reported that 39.3% of participants with best corrected visual acuity worse than 20/40 (or 41 letters in the ETDRS chart) use community or family support [12]. Others found that 36% of AMD patients use paid or unpaid assistance [9]. The estimated intensity of use (amount of caregiver hours) per-week per individual in our sample was 9 hours. This value is between the 4.7 and the 17.4 reported in other studies [9, 11].

The use of informal care was affected by marital status and comorbidities but not by gender. Non-married participants were more likely to report informal care, this may be because they cannot rely on their partners and the need to ask for help is more clearly defined for them. Those married not reporting care may be because, although they rely on their partners for some tasks, they see any help received as a natural gesture of mutual help between members of a couple. It is also intuitive that those with other comorbidities face further difficulties in their daily life and therefore are more likely to require informal care. In a univariate analysis gender seemed to be a predictor but that effect disappeared in the logistic regression. This result is in contrast with some studies reporting that women are more likely to use informal care [12, 13]. It is know that informal care can be influenced by many factors and, in particular, by the organization of the society [5]. Therefore, our results need to be interpreted in the Portuguese context and may also be applicable to similar societies [28].

Visual ability was the only independent predictor of the intensity of use of informal care after adjusting for age, gender and severity of vision impairment. The association between visual ability and informal care shows a link between self-reported task difficulties and the amount of help needed. Others reported an effect of visual acuity; however, these studies did not considered self-reported levels of visual ability [10, 13, 29, 30]. We recognize that visual acuity gives a partial measure of visual performance and that the inclusion of a broader spectrum of visual tests, such as contrast sensitivity or visual field, could provide a better understanding of the association between visual performance and informal care. This would be particularly true in cases of impaired vision caused by diseases such as retinitis pigmentosa in which acuity is preserved but severe functional limitations are imposed by restricted visual fields. In line with our results, Wang et al. [12] found an increase in intensity with increased self-reported walking difficulties. Keefe et al. [26] reported that people with impaired vision need help for vision-dependent activities such as driving, reading documents and support for independent activities outside home. These are the tasks covered by the activity inventory to determine visual ability. In short, visual rehabilitation tailored to increase visual ability is likely to reduce simultaneously the use of informal care and its intensity. These findings suggest that the use of instruments such as the AI during clinical assessment would help to target resources at those with the greatest caring needs.

One of the methodological dilemmas of this study was how to collect information about informal care. We used a questionnaire, the most common method, with a short recall time period to minimize recall bias [5, 25]. Informal care is a frequent event and is known that, contrary to unusual events such as an inpatient stay at a hospital, short recall periods increase the accuracy of the reports [22, 31]. Some participants may, accidentally, extend or reduce recall periods or we might have collected data during seasonal changes in the needs of informal care; however, this factors are unlikely to lead to systematic error in our estimation [31].Other factors, such as treatments or disease changes can alter visual acuity–a 2 week recall period may be beneficial to capture the informal care needed according with the acuity that we measured. Nevertheless, using a short recall period may result in an underestimation of the use of informal care [22]. Keeping a diary would minimize this limitation but would be significantly more expensive and time consuming [5, 32]. In addition, the amount of missing data and the complexity of the information available may increase substantially with a diary [33, 34]. Thus, the method adopted could have led to a conservative estimation of informal care usage. Conversely, to estimate the economic impact of informal care we used opportunity costs which can inflate the costs because it includes 6 caregivers that were retired. The method and value used in our study was used previously in various analysis [5, 10, 25, 26] and is within the interval used in studies using the opportunity cost method [5, 27]. Therefore, it seems appropriate and ensures comparability with others studies [10, 26].

Our participants were recruited at hospitals and therefore our sample includes only patients seeking eye care. Considering that informal eye care is used by patients who are under treatment or have stable eye diseases [5, 7], we believe that the results reported can be generalized to all the Portuguese population with vision impairment. However, information about the characteristics of the Portuguese population with impaired vision is lacking and therefore there is no evidence to confirm this generalization. In addition, we compared the profile of patients who responded our questionnaire and those that declined participation and found some differences. For instance, we found that participation in our study was influenced by gender, distance to the hospital, number of years of education, number of visits to the hospital per year, marital status and visual acuity. This means that the profile of our participants was different from those declining participation.

In summary, this study provides a comprehensive analysis of use of informal care in persons with impaired vision. In the context of the reviewed literature, this study is the first to show a strong link between self-reported ability and the use of informal care in large multi-centre study in an European country. Visual ability was a predictor of the use of informal care and the intensity of care. Therefore, visual rehabilitation interventions, alongside with usual eye care may reduce the economic burden of visual loss at personal and societal level.

## Supporting information

S1 Appendix List of comorbidities. (DOCX)

S2 Appendix Regression models description. (DOCX)
