# Supplementary material for: The use of informal care by people with vision impairment
Source: PLoS One. 2018 Jun 7;13(6):e0198631. doi: 10.1371/journal.pone.0198631 (PMC5991749; doi:10.1371/journal.pone.0198631)
Supplement: S1 Appendix — (DOCX) [file pone.0198631.s001.docx]

# List of comorbidities

| 1. Cancer |
| --- |
| 2. Diabetes |
| 3. Heart condition |
| 4. Hypertension |
| 5. Musculoskeletal disorder |
| 6. Pulmonary disease |
| 7. Stroke or brain hemorrhage |
| 8. Hearing impairments |
| 9. Thyroid condition |
| 10. Psychological problems |
| 11. Neurologic problems |
| 12. Chronic allergies |
| 13. Gastrointestinal condition |
| 14. Liver disease |
| 15. Autoimmune diseases |
| 16. Endocrine condition |
