# Supplementary material for: The use of informal care by people with vision impairment
Source: PLoS One. 2018 Jun 7;13(6):e0198631. doi: 10.1371/journal.pone.0198631 (PMC5991749; doi:10.1371/journal.pone.0198631)
Supplement: S2 Appendix — (DOCX) [file pone.0198631.s002.docx]

# Regression models description

**Logistic regression** - to finds determinants of informal care utilisation. The dependent variable was informal care use (non-user=0; user=1) and the independent variables were: gender (dummy variable: Female= 0; Male =1), marital status (dummy variable: Married= 0; Not Married=1), presence of other comorbidities (more than one comorbidity =0; no comorbidities = 1), visual ability (measured by AI), visual acuity in better eye (logMar scale). The graphic method was used to validate model assumption for residuals independence and to identify extreme cases that were removed from the model whenever it increased the goodness of fit of the model. Multicollinearity was analyzed with Variance Inflation Factor (VIF).

**Multiple linear regression** - to find predictors of informal care utilisation intensity. Depend variable was hours of informal care. Independent variables were visual ability, age, gender (dummy variable: Female= 0; Male =1), and severity of visual impairment transformed into a dummy variable (visual acuity in the better eye equal or below 1 logMar = 0; visual acuity in the better eye above 1 logMar = 1). The model assumption for normality and homoscedasticity of residuals were tested using the Kolmogorov-Smirnoff and the graphic method. Multicollinearity was analysed with Variance Inflation Factor (VIF).
